# Epidemiology, Clinical Features and Treatment of Neurosarcoidosis in Northern Spain

**DOI:** 10.3390/biomedicines13061360

**Published:** 2025-06-01

**Authors:** Alba Herrero-Morant, Raúl Fernández-Ramón, Diana Prieto-Peña, José Luis Martín-Varillas, Santos Castañeda, Ricardo Blanco

**Affiliations:** 1 Rheumatology, HUM Valdecilla, Immunopathology Group, Instituto de Investigación Marqués de Valdecilla (IDIVAL), 39011 Santander, Spain; 2 Ophtalmology, HUM Valdecilla, Immunopathology Group, Instituto de Investigación Marqués de Valdecilla (IDIVAL), 39011 Santander, Spain; 3 Rheumatology, Hospital De Laredo, 39770 Laredo, Spain; 4 Rheumatology, Hospital De La Princesa, IIS-Princesa, 28006 Madrid, Spain

**Keywords:** epidemiology, neurosarcoidosis, incidence, clinical phenotypes, extrapulmonary involvement, treatment, prognosis, biological therapy, autoinmunity, inflammation, sarcoidosis

## Abstract

**Objectives:** Neurosarcoidosis (NS) is a severe and infrequent complication of sarcoidosis. Available data on NS are variable. We aimed to characterize NS epidemiology, clinical and therapeutic characteristics in a well-defined cohort of NS patients. **Methods:** Observational population-based cohort study of 342 patients diagnosed with sarcoidosis in Northern Spain, between 1999 and 2019. Among them, those patients who fulfilled the Consortium Consensus Group diagnosis criteria for NS were included. The annual incidence between 1999 and 2019 was estimated by gender, age, and year of diagnosis. Additionally, a literature review was performed. Therapeutic efficacy was evaluated using the neurological-related extra-pulmonary physician organ severity tool (ePOST). **Results:** NS was diagnosed in 29 out of 342 patients with sarcoidosis (8.5%; 18 women/11 men) with a mean age of 42.3 ± 15.1 years. Most NS patients have associated systemic sarcoidosis (93.4%) mainly consisting of lung (*n* = 22; 75.9%), articular (*n* = 15; 51.7%) and/or ocular (*n* = 12; 40%) involvement. The annual incidence of NS during the study period was 1.1 per 1,000,000 people. There is a linear relationship with a weak decrease in age at diagnosis over time. NS was subdivided into chronic headache (*n* = 11; 36.7%), cranial neuropathy (*n* = 7; 24.1%), myelitis (*n* = 4; 13.8%), peripheral neuropathy (*n* = 3; 10.3%), cranial neuropathy with chronic headache (*n* = 3; 10.3%) and aseptic meningitis (*n* = 2; 6.9%). Twenty-five patients (86.2%) received oral glucocorticoids (mean ± SD maximum prednisone dose 49.6 ± 19.4 mg/day). In addition, conventional immunosuppressive drugs were administered to 17 (58.6%) patients and biological therapy to 12 (41.4%) patients. After 12 months of initiating biological therapy, 14 out of 17 patients (82.4%) achieved complete remission, defined as an ePOST score of 0. Severe allergic reaction was observed in only one patient who had received treatment with both Infliximab and Adalimumab. **Conclusions:** The epidemiological, clinical and treatment characteristics of NS in Northern Spain are similar to that of other countries.

## 1. Introduction

Sarcoidosis is a chronic granulomatous disease of uncertain origin characterized by the presence of noncaseating granulomas [1,2]. It primarily affects the lungs and lymphatic system, although it can potentially involve any organ [1,2,3]. Neurosarcoidosis (NS) is a potentially severe complication that can affect the central, peripheral nervous system or both [4,5]. It is frequently associated with a high level of morbidity and mortality, making treatment nearly always essential [6]. The mortality rate is approximately 5%, and one-third experience limited clinical improvement despite treatment [7].

The clinical presentation of NS can range from mild and nonspecific symptoms to severe manifestations, and it often goes undiagnosed [7,8]. In addition, the available data on NS epidemiology exhibit a significant variability among different studies [7]. This variability may be influenced by biases in patient selection, particularly in cohorts primarily centered on pulmonary sarcoidosis and the lack of uniform criteria [9]. In response to this problem, a set of criteria was proposed in 1999 to standardize the diagnosis and improve comparability among studies [10]. The “Neurosarcoidosis Consortium Consensus Group” is an expert panel of physicians experienced in the management of patients with sarcoidosis and NS, engaged in an iterative process to define NS and develop a practical diagnostic approach to patients with suspected NS [11]. Sarcoidosis annual incidence is estimated to be between 0.3 and 0.5, and 11.5 [12]. Among these cases, NS has been reported to occur in 5% to 10% of all sarcoidosis patients [9]. NS diagnosis is often challenging and only correctly considered in 15% of cases at initial presentation [13]. Indeed, it remains a diagnosis of exclusion, with infectious and malignant etiologies recognized as important mimickers [14].

Treatment is not well standardized and is often delayed. Therefore, it frequently results in unfavorable outcomes with high incidence of permanent neurological deficit in many young patients [15]. Glucocorticoids, such as prednisone, are often the first-line therapy [7]. Other immunosuppressive agents, such as methotrexate, azathioprine or mycophenolate mofetil, may be considered as steroid-sparing agents or used in cases where corticosteroids are not well-tolerated [7]. Biological agents like infliximab (IFX) or adalimumab (ADA) may be used in refractory cases [7]. However, the lack of standardized treatment protocols and the limited number of large-scale studies make treatment difficult to outline [16].

Taking all these considerations into account our study aimed to characterize NS epidemiology, clinical and therapeutic characteristics in a well-defined cohort of NS patients from northern Spain, between 1999 and 2019.

## 2. Materials and Methods

### 2.1. Study Design and Data Collection

We conducted a retrospective cohort study of 342 patients diagnosed with sarcoidosis between 1 January 1999 and 31 December 2019. All patients included in this study resided within the municipalities falling under the Santander Health Area at the time of their diagnosis. The diagnosis procedures were carried out exclusively at the Hospital Universitario Marqués de Valdecilla, which serves as the sole healthcare facility within this health area. Patient identification was performed by utilizing several informatic resources within the Cantabrian Health Service’s intranet, including the hospital’s diagnostic coding system, the International Classification of Primary Care (CIAP) used by health centers, and data obtained from different medical departments, namely Dermatology, Internal Medicine, Neurology, Nuclear Medicine, Ophthalmology, Pulmonology, Pathology, Radiology and Rheumatology.

Diagnosis of NS was based on the NS Consortium Consensus Group diagnosis [11]. The diagnosis of NS was established if (i) the clinical presentation and diagnostic evaluation suggest NS, as defined by the clinical manifestations and Magnetic Resonance Imaging (MRI), Computerized Tomography (CT) scan, Cerebrospinal Fluid (CSF), and/or neurophysiologic study findings typical of granulomatous inflammation of the nervous system after rigorous exclusion of other causes [11]. Probable NS was defined as patients with pathologic confirmation of systemic granulomatous disease consistent with sarcoidosis [11]. Definite NS included patients with nervous system pathology consistent with NS [11]. Patients with probable and definite NS were included in this study [11].

### 2.2. Outcome Variables

The following variables were obtained from the medical records: demographic information (age at diagnosis, gender, ethnicity), smoking status, organ involvement determined by the World Association of Sarcoidosis and other Granulomatous disease (WASOG) criteria, neurological manifestations and results from serological tests, biopsies and radiological findings (chest radiograph, thoracic CT or gammagraphy) [17].

Therapeutic efficacy was also evaluated using the neurological-related extra-pulmonary physician organ severity tool (ePOST) [18,19]. The ePOST score is the sum of 17 extra-pulmonary organ activity scores, each ranging from 0 (no activity) to 6 (maximal activity) [18,19]. The neurological-related ePOST score specifically focuses on neurological involvement and ranges from 0 to 6 [18,19]. A multidisciplinary team retrospectively assessed the severity of sarcoidosis involvement, utilizing a 7-point scale (0: not affected to 6: very severely affected) based on symptoms, MRI, neurophysiology studies and CSF findings. Complete remission was defined by an ePOST score of 0. Partial remission was defined by an improvement of at least 1 point of the neurological-related ePOST score. Patients were considered non-responders if no improvement was observed in ePOST score.

### 2.3. Data Collection and Statistical Analysis

Outcome variables were recorded in accordance with a predefined clinical protocol at the center. Data were stored in a computerized database, and a double-check process was carried out to minimize the entry of wrong data.

To estimate the incidence rates, population data were obtained from the Cantabria Health Service’s annual reports (https://www.scsalud.es/memorias access date: 27 December 2022) and the National Statistics Institute (https://www.ine.es/ access date: 29 December 2022). The annual incidence rate was calculated as the number of new NS cases each year, relative to the population at risk for the disease in that year [20].

The results were presented as mean ± standard deviation (SD) for variables with a normal distribution, or as median and interquartile range (IQR) for those with a non-normal distribution. Qualitative variables were expressed as absolute numbers and percentages (%). The nonparametric Mann–Whitney U test was applied for quantitative variables that did not follow a normal distribution. We calculated Pearson’s correlation coefficient to assess variations in annual incidence and age at diagnosis over time. Confidence intervals (CI) for incidence rates were estimated assuming a Poisson distribution. All statistical analyses were performed using IBM SPSS Statistics for Windows, version 20.0 (IBM Corp., Armonk, NY, USA), and Stata 16/SE (Stata Corp., College Station, TX, USA).

## 3. Results

### 3.1. Demographic Data

All demographic and clinical features are summarized in Table 1. NS was detected in 29 of 342 (8.5%) patients with sarcoidosis. A total of 18 (62.1%) women and 11 (37.9%) men with a mean age at diagnosis of 42.3 ± 15.1 years were included in this study. All patients were Caucasian (*n* = 27; 93.1%) or Hispanic (*n* = 2; 6.9%). Only seven (24.1%) patients smoked.

**Figure 1:**
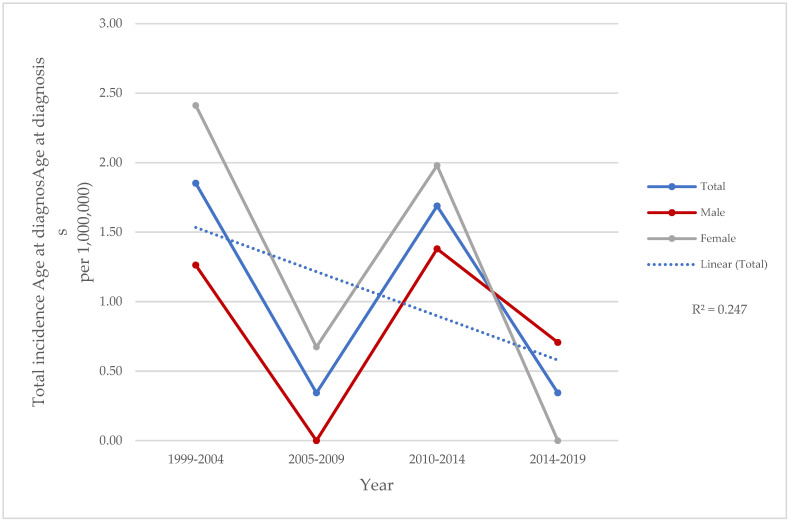
Incidence of neurosarcoidosis in residents of Santander Healthcare Area, Spain, in 1999–2019 according to gender distribution.

The annual incidence of NS in Cantabria during the 1999–2019 period was 1.1 per 1,000,000 people, 95% CI: 1.1–2.6 (0.8 per 1,000,000 men [95% CI: 0.7–2.3] in men, 1.3 per 1,000,000 women [95% CI: 0.9–2.4] in women). There were variations in annual incidences, with a minimum value of 0.8/1,000,000 population in 2013–2014 and a maximum of 1.9/1,000,000 population in 1999–2000 (Figure 1).

**Figure 2:**
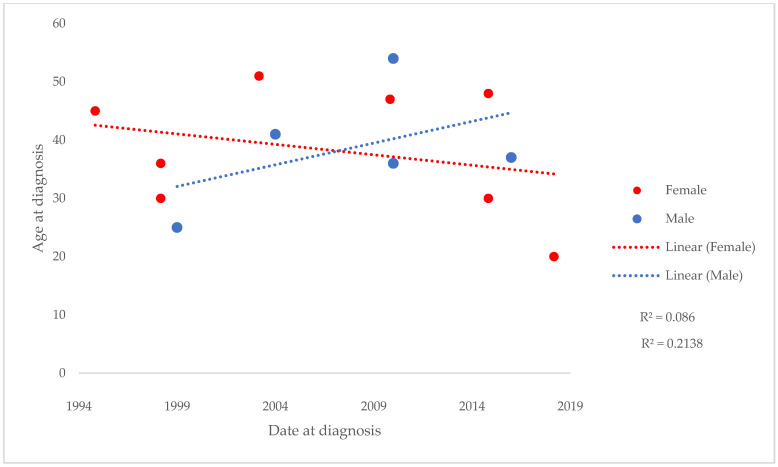
Age trends at neurosarcoidosis diagnosis in Santander Health Area, Spain, in 1999–2019 by gender.

There is a linear relationship with a weak decrease in age at diagnosis over time. Nevertheless, a moderate correlation was found in the case of males (R^2^ = 0.2138) and a weak correlation in the case of females (R^2^ = 0.086) (Figure 2).

### 3.2. Clinical Features at Baseline

The most common neurological manifestation at NS diagnosis was chronic headache (*n* = 10; 34.5%). Headache attributed to neurosarcoidosis was defined based on a modified version of the International Classification of Headache Disorders (ICHD-3) [21]. The criteria for this classification were as follows: (A) Any headache fulfilling criterion C. (B) Sarcoidosis has been diagnosed. (C) Evidence of causation demonstrated by at least two of the following: (i) Headache has developed in temporal relation to the onset of the sarcoidosis. (ii) Either or both of the following: (a) Headache has significantly worsened in parallel with worsening of the sarcoidosis. (b) Headaches have significantly improved in parallel with improvement in sarcoidosis. (iii) Headache is accompanied by one or more cranial nerve palsies. (D) Not better accounted for by another ICHD-3 diagnosis [21].

Cranial neuropathy and myelitis were reported in 24.1% and 13.8% of patients, respectively. The least common manifestations were peripheral neuropathy (*n* = 3; 10.3%), cranial neuropathy with chronic headache (*n* = 3; 10.3%) and aseptic meningitis (*n* = 2; 6.9%). In addition, cranial neuropathy was subdivided into cranial VII palsy (*n* = 5; 50%), optic neuritis (*n* = 3; 30%) and cranial III palsy (*n* = 2; 20%).

The frequency of neurological manifestations did not show gender-based difference, except for cranial neuropathy that was significantly higher in men (45.5% vs. 10.5%; *p* = 0.029).

### 3.3. Multisystemic Involvement

The clinical domains were divided into pulmonary (*n* = 22; 75.9%), articular (*n* = 15; 51.7%), ocular (*n* = 12; 41.4%), cutaneous (*n* = 10; 34.5%) and digestive (*n* = 4; 13.8%). The frequency of these clinical domains did not significantly differ between men and women except for digestive involvement (16.7% in men and 9.1% in women; *p* = 0.028) (Table 1).

### 3.4. Biopsies, Imaging and Laboratory Findings

Regarding laboratory data, angiotensin-converting enzyme (ACE) levels in serum were obtained in 23 patients being elevated in only 5 (21.7%) patients. Hypercalcemia and hypercalciuria were reported in 3.8% and 4.2% of the patients, respectively. Tuberculin skin test was negative in 22 (88%) of the 25 patients evaluated. Cerebrospinal fluid was performed in 13 (44.8%) patients. The most common finding was inflammatory with lymphocyte predominance (*n* = 7; 53.8%), normal (*n* = 5; 41.7) and inflammatory with neutrophil predominance (*n* = 1; 7.1%).

Among the 28 patients who underwent thoracic radiography, 23 (85.2%) exhibited findings compatible with sarcoidosis. Gammagraphy was conducted for a total of 25 patients, revealing signs of sarcoidosis in 16 (64%) patients. Regarding cranial imaging, 26 (89.6%) underwent MRI, 2 (6.9%) had both MRI and CT scans and 1 (3.5%) received a CT scan only. Most patients (*n* = 19; 65.5%) had normal results followed by meningeal (*n* = 3; 10.3%), spinal cord (*n* = 2; 6.9%), cerebral (*n* = 1; 3.4%), cranial nerve (*n* = 1; 3.4%), cranial nerve and spinal cord (*n* = 1; 3.4%), cerebral and spinal cord (*n* = 1; 3.4%) and spinal cord and meningeal (*n* = 1; 3.4%) involvement. Neurophysiology studies were performed in 10 (34.5%) patients. The results show normal findings (4; 40%), polyneuropathy (2; 20%), encephalopathy (2; 20%), chronic neurogenic pattern (*n* = 1; 10%) and mild loss of motor units (*n* = 1; 10%).

Biopsy was performed in 27 (85.2%) patients. The main biopsied organs were mediastinal nodes (*n* = 10; 43.5%) and lungs (3; 13%) (Table 1).

### 3.5. Treatment Administered

Most patients (*n* = 25; 86.2%) received oral glucocorticoids (mean ± SD maximum prednisolone dose 49.6 ± 19.4 mg/day) and seven (24.1%) IV corticosteroids. In addition, conventional immunosuppressive drugs were administered to 17 (58.6%) patients and 26 biological therapies to 12 (41.4%) patients (Figure S1). In four (13.8%) patients, corticosteroids were the only treatment administered.

**Figure 3:**
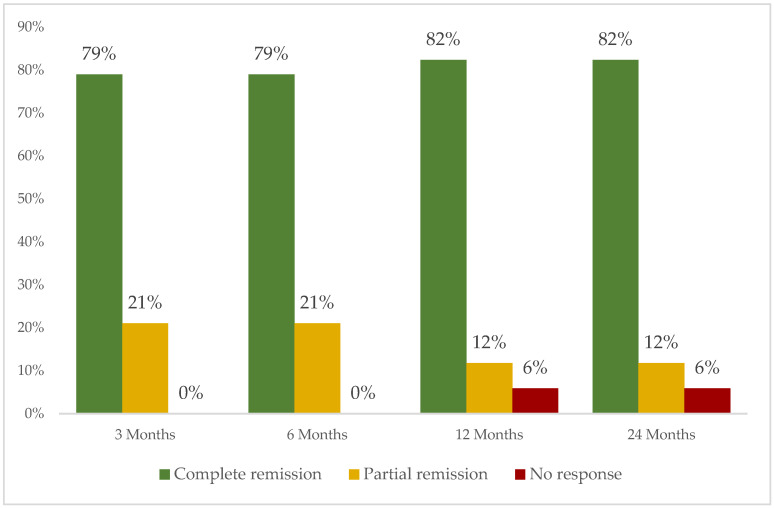
Clinical response of neurosarcoidosis to biological therapy (*n* = 26) in residents of Santander Healthcare Area, Spain, in 1999–2019.

The most frequently used biological therapy was monoclonal tumor necrosis factor inhibitors (anti-TNFα) (*n* = 18; 81.8%) including IFX (*n* = 10; 45.5%) and ADA (*n* = 5; 22.7%) (Figure S1). After 12 months since the initiation of biological therapy, complete, partial or no response was observed in 14 out of 17 (82.4%), 2 (11.8%) and 1 patient (5.9%), respectively (Figure 3). Severe allergic reaction was observed in only one patient who had received treatment with both IFX and ADA. No more severe adverse events were observed. Table 2 shows the treatment according to NS subtypes.

## 4. Discussion

We present the epidemiology, clinical characteristics, and treatment efficacy of NS in a geographically well-defined population. In this study, NS was diagnosed in 8.5% of patients with sarcoidosis, with predominance in women. The annual incidence of NS in our region during the study period (1999–2019) was 1.1 per 1,000,000 people/year. Most patients (93.1%) had associated systemic sarcoidosis. Complete remission after one year since the initiation of biological therapy, was achieved by most patients.

Neurologic manifestations are the initial clinical symptoms in 50–70% of patients with NS, and systemic sarcoidosis is subsequently detected during the diagnostic workup [5]. This highlights the importance of a diagnostic approach that considers both neurological and systemic manifestations.

The main primary studies that analyzed NS epidemiological aspects are summarized in Table 3. The proportion of patients that suffered from neurological complications varies across these studies, ranging from 4.8% in the 2019 USA study to 33.9% in the 2017 French series [5,6,22,23,24,25,26,27]. Our study aligns closely with the findings of the 2011 Spanish study, reporting a percentage of 6.7% [22]. The reasons for these discrepancies in the percentages across studies remain unclear, and one potential factor could be differences in ethnicities. For instance, the 2017 French study indicates a higher percentage of Caucasian participants (53.4%) compared to the 2019 USA retrospective study (3.7%) [24,27]. This ethnic variation has led some studies to postulate the possibility of a higher incidence of NS in Caucasian males [28]. However, conflicting evidence exists, as several studies have found no significant differences in NS incidence between ethnicities [29,30]. The precise reasons for the observed dissimilarities in percentages across studies remain elusive, and further research is needed to elucidate the potential contributing factors.

Regarding gender distribution, most studies, including the current one, indicate a female predominance in NS cases [6,22,23,25]. However, exceptions, are observed in the 2019 USA study (52.4%), the 2022 Belgian study (64%) and the 2022 Danish study (55%) [5,26,27]. These findings align with existing literature suggesting a higher incidence of neurological involvement in females [31]. The potential factors contributing to gender variations in NS incidence are multifaceted [32]. Hormonal, genetic, and environmental influences, along with occupational exposures, medications, smoking habits, and vitamin D deficiency, could collectively contribute to the observed differences [32].

In our study, mean age at diagnosis was 42.3 ± 15.1 years. These findings are similar to the studies examined [5,6,22,23,24,25,26,27]. The age range spans from 31.5 years in the 2017 French study to 51.6 years in the Danish study, deviating from the expected trend outlined in existing literature [16,24,26]. However, NS is typically associated with a predilection for adults around 40.5 years (range 22 to 67 years) [5]. Nevertheless, certain series have established that the mean age at onset is 33 to 41 years [33]. The disparity between the observed age ranges and the established pattern in the literature raises questions about the potential influence of distinct population characteristics, regional factors, or study-specific criteria.

The observed clinical characteristics align with those reported in prior studies [7,13,14]. For example, the prevalence of chronic headache (34.5%), cranial neuropathy (3%) and peripheral neuropathy (10.3%) in our study falls within the previously documented ranges of 32%, 11–55% and 17%, respectively [7,13,14]. However, direct comparisons are challenging due to the absence of a standardized classification of clinical phenotypes in neurosarcoidosis [7,13,14].

In this study, most patients (*n* = 19; 65.5%) had normal MRI results, which is substantially higher than the reported 2% in existing literature [27]. This discrepancy may be attributed to the prevalence of cranial and peripheral neuropathies in this study. It is known that MRI findings can be normal or exhibit minimal correlation with symptoms in cases of neuropathies, even when clinically evident [1].

Regarding treatment, half of the patients in our study needed biological therapy. Twelve months after the initiation of biological therapy, a complete response was observed in 14 of 17 (82.4%). These findings are similar to other series conducted across multicenter studies in the US, Utah, and the Netherlands [9]. In these studies, a favorable clinical response was reported in 77%, 87%, and 96% of patients, respectively [7,34,35]. However, the majority of these studies primarily focus on IFX [15]. These findings are consistent with the 2025 Consensus Recommendations for the Management of Neurosarcoidosis which strongly favor anti-TNF-α inhibitor therapies, particularly IFX, as the preferred steroid-sparing agent during induction [36]. Alternative options such as ADA and other non-anti-TNFα such as rituximab, tocilizumab, and tofacitinib only have limited but successful case series [15,37]. In our series, four patients received non-anti- TNFα: etarnecept (*n* = 1), tocilizumab (*n* = 1), secukinumab (*n* = 1) and rituximab (*n* = 1). Complete or partial remission was achieved in all these patients except with secukinumab. Nonetheless, more studies are needed to establish the effectiveness of these treatments.

The present study has several limitations. First, patients were mainly identified using diagnosis codes from a single referral hospital database. This could potentially lead to selection bias, limit some cases’ detection and therefore underestimate right incidence rates. Second, the retrospective nature of this study could also underestimate the true incidence of this complication. However, the total number of patients with sarcoidosis and NS in our study is within the range expected according to the other studies shown in Table 3. Third, the study’s assessment of treatment response for NS has limitations. The ePOST score, used for defining remission, is subjective and may not capture all neurological change. Fourth, headache attributed to NS was classified according to an adaptation of the ICHD-3 [21]. This modification could lead to an overestimation of headache directly caused by NS, as it risks misclassifying headaches that are merely coincidental or related to general systemic inflammation rather than specific neurological involvement.

## 5. Conclusions

In summary, our results indicate that the epidemiological, clinical and treatment characteristics of NS in Northern Spain are similar to those of other countries. It usually manifests as chronic headache, peripheral neuropathy and/or cranial neuropathy. Cranial neuropathy seems significantly more frequent in men than in women. A high proportion of patients will require biological therapy. In this line, anti-TNF agents are the most used agents.

## Figures and Tables

**Table 1:** Demographic and clinical features of 29 patients with neurosarcoidosis in the 1999–2019 period. Differences according to gender.

Clinical Characteristics	Total (*n* = 29)	Female (*n* = 18)	Male (*n* = 11)	*p*
**Demographic parameters**				
- Age at diagnosis, years (mean ± SD) - Follow-up time, years (mean ± SD) - Caucasian, *n* (%) - Hispanic, *n* (%) - Smokers, *n* (%)	42.3 ± 15.1 11.9 ± 6.6 27 (93.1) 2 (6.9) 7 (24.1)	46 ± 16.1 11.5 ± 6.1 16 (88.9) 2 (11.1) 2 (11.1)	35.7 ± 12.2 12.5 ± 7.7 11 (100) 0 5 (45.5)	0.350 0.174 0.265 0.265 **0.023**
**Non-neurological systemic involvement, *n* (%)**	27 (96.6)	18 (100)	9 (81.1)	0.054
- Pulmonary involvement - Articular involvement - Ocular involvement - Cutaneous involvement - Digestive involvement	22 (75.9) 15 (51.7) 12 (41.4) 10 (34.5) 4 (13.8)	15 (83.3) 11 (61.1) 7 (38.9) 7 (38.9) 3 (16.7)	7 (54.5) 4 (36.4) 5 (26.3) 3 (27.3) 1 (9.1)	0.199 0.256 0.643 0.417 **0.028**
**Neurological involvement, *n* (%)**	29 (100)	18 (100)	11 (100)	0.164
- Chronic headache - Cranial neuropathy - Myelitis - Peripheral neuropathy - Chronic headache and cranial neuropathy - Aseptic meningitis	10 (34.5) 7 (24.1) 4 (13.8) 3 (10.3) 3 (10.3) 2 (6.9)	6 (33.3) 2 (11.1) 4 (22.2) 3 (16.7) 2 (11.1) 1 (5.6)	4 (36.4) 5 (45.5) 0 0 1 (9.1) 2 (18.2)	0.979 **0.029** 0.102 0.165 0.900 0.685
**Laboratory findings, *n* (%)**				
- Negative tuberculin skin test - Elevated serum ACE levels - Hypercalcemia - Hypercalciuria	22/25 (88) 5/23 (21.7) 1/26 (3.8) 1/24 (4.2)	15/17 (81.2) 3/14 (21.4) 0 0	7/8 (87.5) 2/9 (22.2) 1/9 (11.1) 1/8 (12.5)	0.958 0.808 0.197 0.149
**Biopsy, *n* (%)**	23/27 (85.2)	14/17 (82.4)	9/10 (90)	0.629
- Mediastinal nodes - Lungs - Other (salivary gland, nerve, cutaneous…)	10/23 (43.5) 3/23 (13) 10/23 (43.5)	6/14 (42.9) 1/14 (7.1) 7/14 (50)	4/9 (44.4) 2/9 (22.2) 3/9 (33.3)	0.831 0.265 0.341
**Imaging, *n* (%)**				
- Compatible thoracic radiography - Compatible thoracic tomography - Compatible gammagraphy	23/27 (85.2) 23/26 (88.5) 16/25 (64)	14/17 (82.4) 14/16 (87.5) 9/16 (56.3)	9/10 (90) 9/10 (90) 6/9 (66.7)	0.629 0.888 0.696

Abbreviations: ACE: Angiotensin-Converting Enzyme. Bold numbers indicate statistical significance (*p* < 0.05).

**Table 2:** Treatment of 29 patients with neurosarcoidosis here included.

NS Subtype	*N* (%)	Other Clinical Manifestations	Conventional Immunosuppressant, *N* = 22	Monoclonal Anti-TNFα, *N* = 22	ETN, *N* = 1 *N* (%)	TCZ, *N* = 1 *N* (%)	SCK, *N* = 1 *N* (%)	RTX *N* = 1 *N* (%)
Chronic headache	10 (34.5)	Articular (*n* = 7, 70%) Lung (*n* = 6, 60%) Skin (*n* = 3, 30%) Ocular (*n* = 3, 30%) GI (*n* = 2, 20%)	MTX (*n* = 5, 50%) AZA (*n* = 1, 10%)	GLM (*n* = 1, 10%)	0	0	1 (10)	0)
Cranial neuropathy	7 (24.1)	Lung (*n* = 5, 71.4%) Ocular (*n* = 5, 71.4%) Articular (*n* = 3, 42.9%) Skin (*n* = 3, 42.9%) GI (*n* = 1, 14.3%)	MTX (*n* = 5, 71.4%) AZA (*n* = 5, 71.4%)	IFX (*n* = 3, 42.9%) ADA (*n* = 3, 42.9%) GLM (*n* = 1, 14.3%)	1 (14.3)	0	0	0
Myelitis	4 (13.8)	Lung (*n* = 4, 100%) Ocular (*n* = 1, 25%) Skin (*n* = 1, 25%) Articular (*n* = 1, 25%)	MTX (*n* = 2, 50%)	IFX (*n* = 2, 50%) GLM (*n* = 1, 25%)	0	0	0	0
Peripheral neuropathy	3 (10.3)	Lung (*n* = 3, 100%) Ocular (*n* = 1, 33.3%) Articular (*n* = 1, 33.3%)	MTX (*n* = 1, 33.3%) AZA (*n* = 1, 33.3%)	IFX (*n* = 1, 33.3%)	0	0	0	0
Chronic headache and cranial neuropathy	3 (10.3)	Lung (*n* = 3, 100%) Skin (*n* = 3, 100%) Articular (*n* = 3, 100%) Ocular (*n* = 2, 66.7%) GI (*n* = 1, 33.3%)	MTX (*n* = 2, 66.7%)	ADA (*n* = 2, 66.7%) IFX (*n* = 1, 33.3%)	0	0)	0	1 (33.3)
Aseptic meningitis	2 (6.9)	Lung (*n* = 1, 50%)	MTX (*n* = 1, 50%)	IFX (*n* = 1, 50%) ADA (*n* = 1, 50%)	0	1 (50)	0	0
Total (*n* = 29)	29 (100)	Lung (*n* = 22, 75.9%) Articular (*n* = 15, 51.7%) Ocular (*n* = 12, 41.4%) Skin (*n* = 10, 34.5%) GI (*n* = 4, 13.8%)	MTX (*n* = 15, 51.7%) AZA (*n* = 7, 24.1%)	IFX (*n* = 8, 27.6%) ADA (*n* = 6, 20.7%) GLM (*n* = 3, 10.3%)	1 (3.4)	1 (3.4)	1 (3.4)	1 (3.4)

Abbreviations: ADA: adalimumab, AZA: azathioprine, ETN: etarnecept, GLM: golimumab, GI: gastrointestinal, IFX: infliximab, MTX: methotrexate, SCK: secukinumab, RTX: rituximab, TCZ: tocilizumab.

**Table 3:** Main studies analyzing epidemiological aspects of neurosarcoidosis.

Author, Year	Country	Cases			Male, *N* (%)		Age at Onset Years, Mean ± SD	
		S	NS	%	S	NS	S	NS
Gascón-Bayarri et al., 2011 [22]	Spain	445	30	6.7	ND	10 (33.4)	ND	48.3 ± ND
Leonhard et al., 2016 [23]	The Netherlands	ND	52	ND	ND	22 (48.0)	44 ± ND	43.0 ± ND
Joubert et al., 2017 [24]	France	690	234	33.9	ND	117 (50)	ND	31.5 ± ND
Dorman et al., 2019 [27]	USA	1706	82	4.8	691 (40.6)	43 (52.4)	49 ± 10.8	45.0 ± 11.4
Arun et al., 2020 [6]	UK	ND	80	ND	ND	35 (44)	ND	47.8 ± ND
Goel et al., 2020 [25]	India	ND	12	ND	ND	4 (33.4)	ND	44.0 ± 9.2
Sambon et al., 2022 [5]	Belgium	180	22	12.2	ND	14 (64)	ND	40.5 ± ND
Byg et al., 2022 [26]	Denmark	ND	20	ND	ND	11 (55)	ND	51.6 ± ND
Present study, 2025	Spain	342	29	8.5	165 (48.2)	11 (37.9)	47.7 ± 15.1	42.3 ± 15.1

Abbreviations: ND: non data available, NS: neurosarcoidosis, S: sarcoidosis.

## Data Availability

The original contributions presented in this study are included in the article, further inquiries can be directed to the corresponding author.

## Supplementary Materials

The following supporting information can be downloaded at: https://www.mdpi.com/article/10.3390/biomedicines13061360/s1, Figure S1: Flow-chart of conventional and biologic therapies used in patients with neurosarcoidosis.
