# Supplementary material for: Epidemiology, Clinical Features and Treatment of Neurosarcoidosis in Northern Spain
Source: Biomedicines. 2025 Jun 1;13(6):1360. doi: 10.3390/biomedicines13061360 (PMC12190128; doi:10.3390/biomedicines13061360)
Supplement: Supplementary file 1 [file biomedicines-13-01360-s001.zip › biomedicines-3610752-supplementary.pdf]

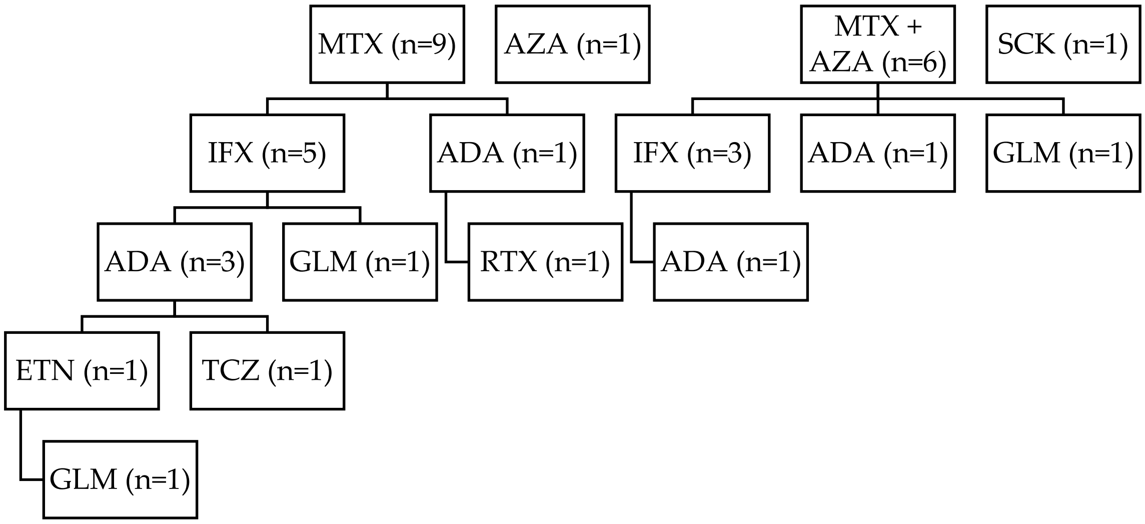

Abbreviations: ADA: Adalimumab, AZA: Azathioprine, ETN: Etarnecept, GLM: Golimumab, GI: Gastrointestinal, IFX: Infliximab, MTX: Methotrexate, SCK: Secukinumab, RTX: Rituximab, TCZ: Tocilizumab

Figure S1. Flow-chart of conventional and biologic therapies used in patients with neurosarcoidosis
